# Age-related changes in trunk muscle activity and spinal and lower limb kinematics during gait

**DOI:** 10.1371/journal.pone.0206514

**Published:** 2018-11-08

**Authors:** Rebecca Crawford, Leonardo Gizzi, Angela Dieterich, Áine Ni Mhuiris, Deborah Falla

**Affiliations:** 1 Institute for Health Sciences, Zürich University of Applied Sciences, Winterthur, Switzerland; 2 University Medical Center Göttingen, Göttingen, Niedersachsen, Germany; University of Florida, UNITED STATES

## Abstract

The influence of age on spinal muscle activation patterns and its relation to kinematics is poorly understood. We aimed at understanding age-related changes to spine and trunk muscle activity in addition to spinal and lower limb kinematics during treadmill walking under various conditions. An observational study was conducted evaluating asymptomatic young (n = 10; 3F, 7M; 26.3±2.5yrs) and older (n = 9; 3F, 6M; 67.1±4.2yrs) adults’ treadmill walking at 2km/h and 4km/h, each at 0, 1, 5, and 10% inclination. Unilateral (right side) electromyography (EMG) was recorded from deep and superficial multifidus (intramuscular) and erector spinae and abdominal obliques (surface); trunk and leg kinematics were also measured. Muscle activity was characterised by peak amplitude and duration of activity, and the time-point of peak amplitude in the gait cycle (0–100%). Peak activation in older adults was lower for the superficial multifidus (p<0.0001) and higher for the thoracolumbar (p<0.001) and lumbar erector spinae (p<0.01). The duration of activation was longer in older adults for all muscles (p<0.05) except the superficial multifidus, and longer during faster walking for all participants. The time-point of peak amplitude in the gait cycle was earlier in older participants for the external obliques (p<0.05). Walking speed appeared to influence muscle activity more than inclination. Older adults used less spine, trunk and lower limb motion, except at the ankle. Age-related differences within multifidus and between paravertebral and trunk muscles were inconsistent. Walking at 4km/h at 5–10% inclination may specifically target the lumbar paravertebral muscles.

## Introduction

Walking is a fundamental human ability essential for social participation that is widely promoted as being beneficial to health in both young and older adults. Walking is often trained in rehabilitation, including for the management of spine-related conditions. Yet, surprisingly little evidence supports walking or gait-based activities as interventions specifically directed at the spine. Further, little is known regarding the activation of spinal muscles during walking, particularly for the deep-lying muscle fibres. Momentum is mounting toward research of spinal muscles in understanding spinal degeneration, pain, function, and recovery. However, age is a strong confounder [1–4] that confuses the clinical significance of muscle parameters [5–7]. As far as we are aware, no study has directly examined the influence of age on activity of the muscles of the lumbar spine, or trunk kinematics during walking.

Change to gait patterns and muscle activity occur with ageing [8, 9]. Older adults walk more slowly [8–11], have wider and shorter stance [10–14] and swing phases [14], and exhibit reduced range of motion of the lower limb [15, 16] than younger adults [17]. However, these findings are informed by gait kinematic studies^.^[8–10, 12–19] or studies examining young adults^.^[10, 20–26]. One study reported an incremental reduction in co-activation of the trunk muscles with age, and increased coactivation with gait speed [10]. However, they did not detail age-related differences according to individual muscles or relating to gait kinematics.

Multifidus has received considerable attention as an influential muscle in the lumbar spine; it has a mixed function [27] based on a complex morphology [28, 29]. Fibres that are closest to the lamina and axis of rotation are shortest, span two motion segments, and are thought to support the motion segment through a predominantly stabilising role [27, 30]. Relatively superficial multifidus fibres span up to four segments and produce phasic motion including trunk extension [27–29]. While an early report suggested a differential fibre type distribution within multifidus [31], it is contemporarily held that the regional distribution of fibre types is consistent among the posterior paravertebral muscles [32]. Intramuscular EMG (iEMG) studies examining sagittal plane motion showed differential activation within multifidus [23, 26, 33], with similar potential for the thoracolumbar erector spinae [34–36]. However, voluntary differential activation is challenging for many people [37, 38] and it is unclear whether differential activation of the spinal muscles can be integrated into rehabilitation. As such, identifying methods for activating these muscles within common activities of daily living, like walking, may be valuable.

Faster treadmill-walking (~4 km/hr) at a higher incline (~5–15%) has been shown to preferentially activate lumbar paravertebral muscles in young adults [21, 26], which may represent walking conditions to target these morphologically complex muscles [28, 29]. Weber et al [25] indicated higher activation of multifidus in young adults during a challenging treadmill walking condition compared to self-selected ‘normal’ treadmill walking, and also showed differential activation in deep and superficial trunk muscles. Further, Mazaheri et al [22] showed higher trunk and spine muscle amplitudes for treadmill walking than over-ground walking, and Belavy et al [39] reported superior intervertebral disc quality after fast walking and slow running over-ground, but not after higher impact tasks, low intensity walking or static positions. While emerging evidence supports the concept that gait-based exercise interventions are potentially beneficial to specific spinal tissues like muscles [22, 25, 39, 40], whether this is equivalently so for young and older adults is unclear.

It is plausible that differences in lumbar paravertebral muscle activation exist between young and older healthy adults during walking. Muscle activation may also be influenced by adaptation of posture and trunk kinematics used to accommodate different walking conditions. Using an observational design, our main aim was to determine the activation of deep and superficial multifidus (using iEMG), thoracolumbar erector spinae and abdominal external and internal obliques (using surface EMG; sEMG) in young and older adults during treadmill walking under different speed-inclination conditions. Further, we concurrently examined spine and lower limb kinematics. We hypothesised that activation within the paravertebral and oblique muscles would be higher in older adults during faster walking and at higher inclines secondary to increased demands on neuromusculoskeletal motor control. Further, we expected the older participants to use less range of motion throughout the spine and lower limb. Finally, we hypothesised that faster walking and/or higher inclination would result in greater muscle activation for both age groups, and that superficial and deep multifidus fibres would be differentially activated.

## Materials and methods

### Participants

Ten asymptomatic adults in their twenties (three women, seven men; aged 26.3±2.5 years; BMI 23.5±4.5 kg/m^2^) and nine asymptomatic older adults aged in their 60s or 70s (three women, six men; aged 67.1±4.2 years; BMI 25.0±2.9 kg/m^2^) participated. Volunteers with current or previous (12 months) LBP that required treatment, where LBP was defined as pain in the low back and/or lower leg (sciatica), were excluded. Other exclusions included musculoskeletal injury/disorder, cardiovascular or neurological disorders, diabetes, previous infections following clinical needle insertion, coagulation disorders, medications affecting such, or unfamiliarity with treadmill walking. Recruitment was achieved through local advertisements at the university and large teaching hospital. Approvals were achieved from the University Medical Centre Göttingen Ethics Committee, and complied with the Declaration of Helsinki. Written informed consents prior to participation were obtained; participants were nominally remunerated.

### Intramuscular electromyography (iEMG)

According to the method of MacDonald *et al*. [41], the skin was disinfected (injection swabs: 70% isopropylalkohol, 30x30 mm, Selefatrade, Spånga, Sweden) and wire electrodes comprising Teflon-coated stainless steel (diameter: 0.1 mm; A-M Systems, Carlsborg, WA) were inserted under ultrasound guidance (Echo Blaster, Telemed; 10-MHz linear transducer) into the right deep and superficial regions of the multifidus at the level of L5 via 27-gauge hypodermic needles. Approximately 3–4 mm of tip-insulation was removed to obtain an interference EMG signal. Separate needles were inserted into the deep (medial approximating the spinous process-lamina junction) and superficial (lateral and deep to subcutaneous tissue) multifidus fibres and removed immediately to leave the wires in the muscle for the duration of the experiment. Signals were acquired in monopolar mode. Reference electrodes were placed over the right iliac crest and posterior superior iliac spine (PSIS).

### Surface electromyography (sEMG)

The skin was prepared by gentle local abrasion (Medic-Every, Parma, Italy) and cleansed with water. Bipolar surface electrodes with an inter-electrode distance of 20mm were placed according to published guidelines [42] and confirmed by manual muscle testing. A reference electrode was placed over the iliac crest. Electrode locations were:

1. Three thoracolumbar ES (longissimus) sites visually located in series approximately 30mm right of the spinous processes: Lumbar—peak of the lumbar curvature (~L3); thoracolumbar junction—level of curvature inflexion; and thoracic—peak of thoracic kyphosis (~T6/T7).
2. External abdominal oblique (EO): immediately inferior to the anterior angle of the eighth/ninth rib (angled at ~45°).
3. Internal abdominal oblique (IO): Medial to the anterior superior iliac spine (ASIS; horizontal orientation).

EMG data was band pass filtered (8th order zero lag band pass 10-500Hz), sampled at 2048 samples/s, 12-bit A/D converted (EMG-USB2, OT Bioelettronica, Turin, Italy) and saved on a personal computer HDD (OTBiolab software V.2.05, OT Bioelettronica, Turin, Italy).

### Motion capture

Tridimensional tracking of gait cycles was achieved using an eight-camera stereo-photogrammetry system (Oqus 300+, Qualisys Gothenburg, Sweden). Retro-reflective markers were placed on each subject’s skin overlying the following visibly-detected landmarks: seventh cervical spinous process (C7), peak of the thoracic kyphosis (TK), thoracolumbar junction (~T11; TLJ), peak of the lumbar lordosis (~L3; LUM), sacrum (~S2; SACR), and bilateral PSISs at a bilaterally equivalent level and slightly superior to the iEMG reference electrode (on the right), greater trochanters, fibula heads, lateral malleoli, and fifth metatarsals. Kinematic data was sampled at 256 frames/s (Qualisys Track Manager V. 2.8, Qualisys AB Gothenburg, Sweden) together with one analog channel for synchronization (described below).

### Procedure

Participants were given time to familiarise themselves with treadmill walking at a self-selected speed and at different inclinations. An investigator controlled the treadmill settings, explained the procedure, and remained in close proximity to the participant to optimise safety. Participants were required to walk continuously for two minutes at 2km/h and then 4km/h, and at each of 0, 1, 5, and 10% inclination undertaken in an incremental order at slow and then faster speed; 30-45s rest was given (at 0km/h-0%) between each test condition. Data was captured for the last 90s for each trial.

### Data analysis

Motion capture and EMG data were synchronized by mirroring the deep multifidus EMG channel on the A/D converter (digitized at 2048sample/second, 12-bit depth) and offline-computing the time delay as the maximum of the cross-correlation function of the two signals [43]. To account for differing stride cadences, and in order to analyse the same stride number per condition, the 41 middle gait cycles (i.e. the middle, previous, and following 20 gait cycles) were retained for further analyses. Analyses were performed through custom-written Matlab scripts (Matlab2013a, Mathworks, Natick, USA).

Kinematic data was low-pass (zero-lag) filtered (10Hz, 2^nd^-order Butterworth filter) according to previously reported methods [44]. Individual gait cycles were identified as two consecutive heel strikes; briefly: right heel strikes were identified as the local maxima (i.e. the sample where the sign of the first derivative of the signal changed from positive to negative) of the sagittal component of the right ankle marker; each gait cycle was time-interpolated to obtain a constant length of 200 samples independently on the time duration [45]. Across each gait cycle, ankle, knee, hip, lumbar lordosis (defined by SACR, LUM, and TLJ markers), and trunk inclination (C7-SACR line versus vertical) (sagittal plane), and pelvis rotation (transverse plane) range of motion (ROM) were computed. The pelvis was modelled as a rigid body, and rotation in the transverse plane (left to right) used for analysis [46]. Each ROM was calculated for each gait cycle as the maximum minus the minimum angles, reflecting average motion excursion for each trial. Hip, knee and ankle angles were averaged for both sides.

The EMG envelope (rectified and low-pass filtered as described above) was segmented according to the previously identified heel strikes, and time-interpolated to 200 samples. For each muscle, activation was characterized by: amplitude of the peak of activation (normalized to maximal activity at 2km/h-0%), duration of the muscle activation computed as the percentage of the gait cycle exceeding an activity threshold, and position of the peak of activation within the gait cycle (%). The latter was computed as follows: the gait cycles were divided in 20 intervals (5% duration each); for each interval average and standard deviation of the EMG envelope were computed. The threshold was set as the average plus three times the standard deviation of the interval with the lowest average value [26, 47]. Peak amplitude, activity duration, and peak position were computed for each of the 41 gait cycles per condition, and average values were retained for statistical analysis.

### Statistical analysis

Peak EMG amplitude were analysed with two-way analysis of variance (ANOVA) for each muscle region (multifidus (superficial and deep), thoracolumbar ES (lumbar, thoracolumbar junction, thoracic), abdominal obliques (external, internal)) with group (young, older) and condition (2km/h-1%, 2km/h-5%, 2km/h-10%, 4km/h-0%, 4km/h-1%, 4km/h-5%, 4km/h-10%) as factors. Percentage of the gait cycle where peak EMG amplitude was detected, and duration of muscle activation expressed as a percentage of gait cycle, were analysed using three-way ANOVA with group, speed (2km/h, 4km/h), and inclination (1%, 5%, 10%). Lumbar lordosis, trunk inclination, pelvic rotation and hip, knee and ankle range of motion were analysed with three-way ANOVA with group, speed and inclination. Significant differences revealed by ANOVA were followed by Student-Newman-Keuls (SNK) pair-wise comparisons. Statistical analyses were performed with SPSS Version 22.0 (IBM, Armonk, USA). Statistical significance was p<0.05.

## Results

### Muscle activation

#### Deep multifidus

**Fig 1:**
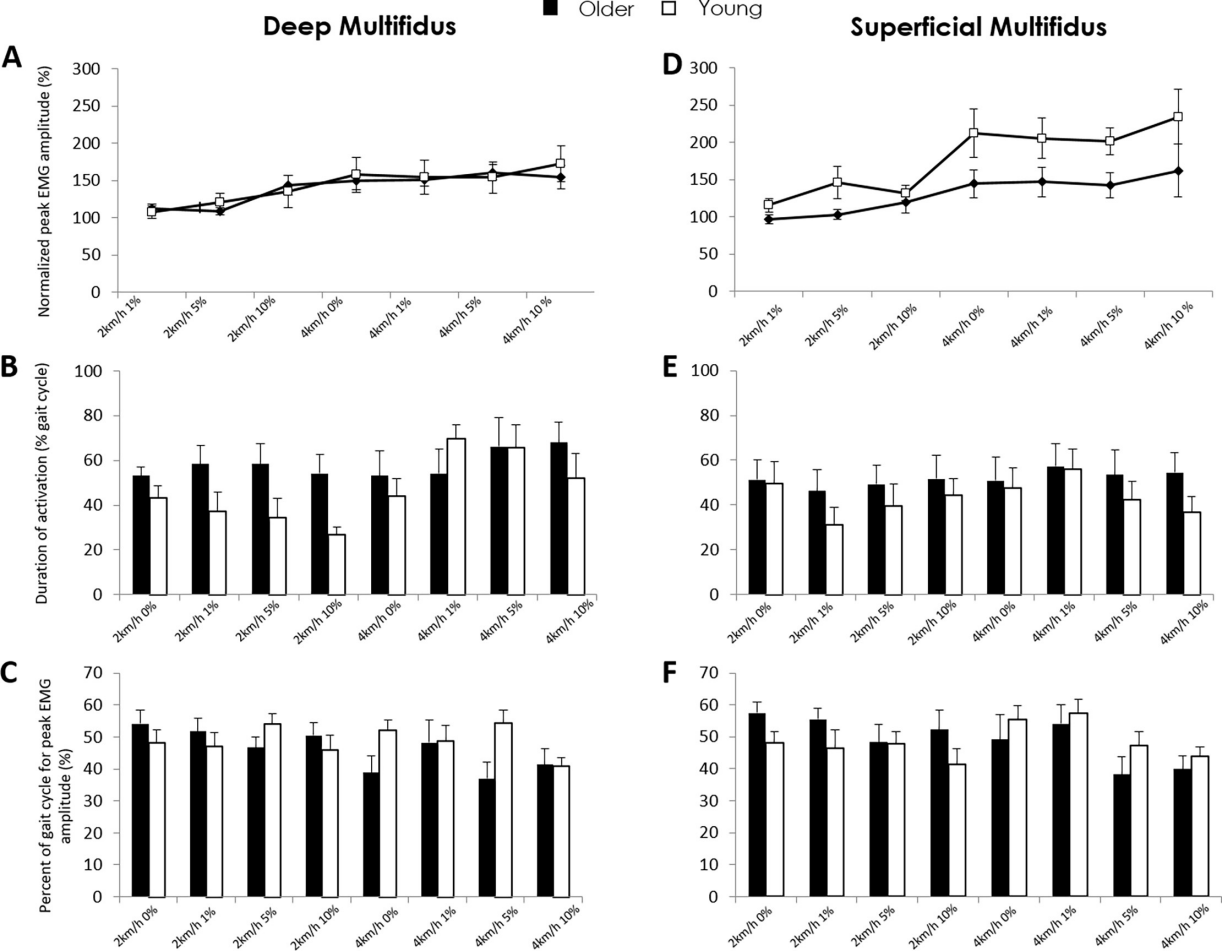
Intramuscular EMG amplitude (mean and SD) in young and older adults for deep (left) and superficial (right) multifidus during treadmill-walking under various speed-inclination conditions. A&D, Normalised (to 2km/h-0%) peak amplitude; B&E, Duration of activation as percent of the gait cycle; C&F, Percentage of the gait cycle where peak amplitude occurred.

Peak EMG amplitude did not differ between groups but depended on condition (F = 3.19, p<0.01); amplitude of activation was higher at 4km/h-10% inclination than 2km/h-1% & 2km/h-5% (SNK: p<0.05) (Fig 1A). Duration of activation was dependent on group (F = 6.54, p<0.05), speed (F = 8.94, p<0.01) and interaction between group and speed (F = 4.05, p<0.05). Deep multifidus duration was longer when walking at faster speed (all inclinations) in young adults (p<0.01), and did not change with speed in older adults. Deep multifidus activation was longer for the older group when walking at 2km/h (p<0.01) (Fig 1B). The percentage of gait cycle when peak deep multifidus amplitude was detected was dependent on interaction between group and speed (F = 4.45, p<0.05); occurred earlier in the gait cycle at 4km/h than 2km/h for older adults but remained the same for the young (SNK: p<0.01). Peak activity occurred earlier in the gait cycle in older participants at 4km/h for each inclination (SNK: p<0.05) (Fig 1C).

#### Superficial multifidus

Peak amplitude was dependent on group (F = 15.68, p<0.0001) and condition (F = 4.77, p<0.0001) but not the interaction. Superficial multifidus activity was lower in the older participants across all walking conditions (SNK: p<0.001). For both groups, peak amplitude was greater for all inclinations at 4km/h than 2km/h (Fig 1D). Duration of activation did not differ between groups or with speed or inclination (Fig 1E). Percentage gait cycle where peak superficial multifidus (iEMG) amplitude occurred did not differ between groups, or with speed or inclination (Fig 1F).

#### Lumbar erector spinae

**Fig 2:**
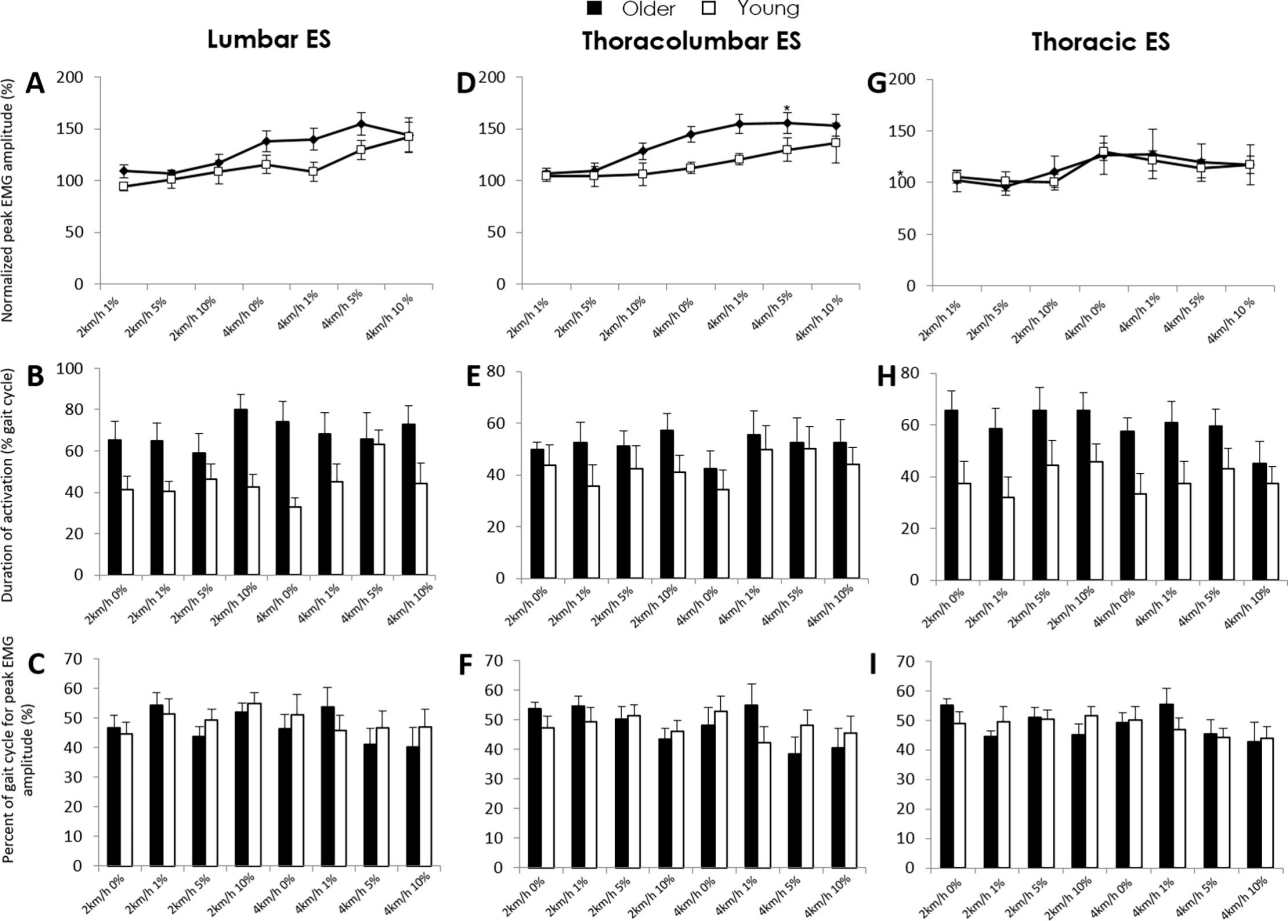
Surface EMG amplitude (mean and SD) in young and older adults for lumbar (left), thoracolumbar (centre) and thoracic (right) erector spinae during treadmill-walking under various speed-inclination conditions. A&D, Normalised (to 2km/h-0%) peak amplitude; B&E, Duration of activation as percent of the gait cycle; and C&F, Percentage of the gait cycle where peak amplitude occurred.

Peak amplitude was dependent on group (F = 7.72, p<0.01) and condition (F = 5.09, p<0.001) but not their interaction. Peak lumbar erector spinae activation was greater for older participants across all conditions (SNK: p<0.01) and greater when walking at 4km/h-5%&10% inclination compared to walking at 2km/h at each inclination (SNK: all p<0.05) (Fig 2A). Duration of activation was longer for older participants (F = 31.14, p<0.00001; SNK: p<0.00001) (Fig 2B). The percentage gait cycle where peak lumbar erector spinae (sEMG) amplitude occurred did not differ between groups or with speed or inclination (Fig 2C).

#### Thoracolumbar erector spinae

Peak amplitude was dependent on group (F = 13.17, p<0.001) and condition (F = 5.09, p<0.001) but not their interaction. Peak activation of thoracolumbar erector spinae was greater in older adults across all conditions (SNK: p<0.001) and when walking at 4km/h compared to 2km/h-1%&5% (SNK: all p<0.05) (Fig 2D). Duration of thoracolumbar erector spinae activation was longer for older participants (F = 5.13; SNK: p<0.05) but unchanged with speed and inclination (Fig 2E). The percentage gait cycle where peak thoracolumbar erector spinae (sEMG) amplitude occurred did not differ with group, speed or inclination (Fig 2F).

#### Thoracic erector spinae

Peak thoracic erector spinae amplitude also did not differ between groups or with speed or inclination (Fig 2G). However, the duration of thoracic erector spinae activity was longer for older participants (F = 27.34, p<0.000001; SNK: p<0.000001) (Fig 2H). The percentage gait cycle where peak thoracic erector spinae (sEMG) amplitude occurred did not differ between groups or with speed or inclination (Fig 2I).

#### Internal oblique

**Fig 3:**
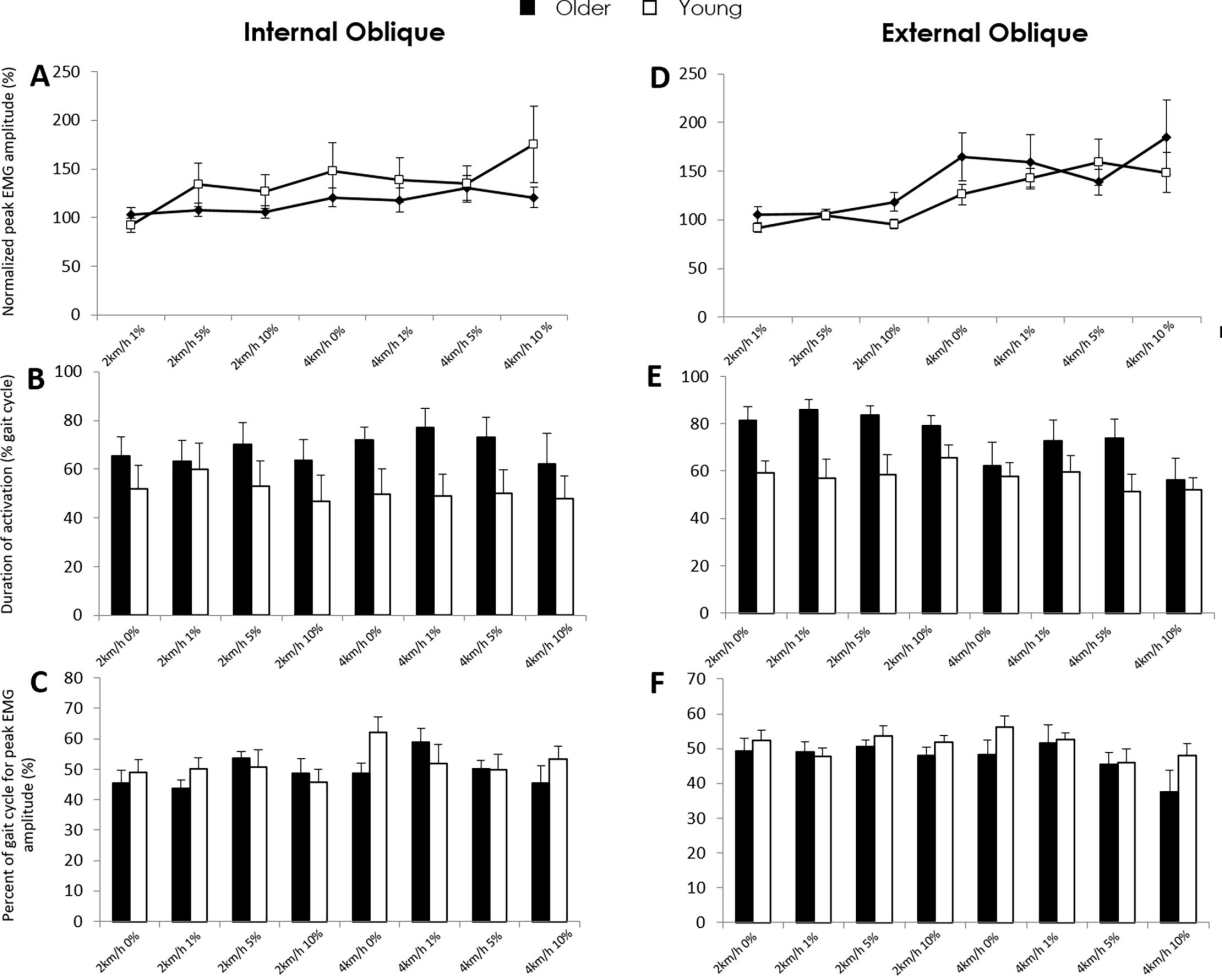
Surface EMG (means with SDs) in young and older adults for internal (left) and external (right) obliques during treadmill-walking under various speed-inclination conditions. A&D, Normalised (to 2km/h-0%) peak amplitude; B&E, Duration of activation as percent of the gait cycle; and C&F, Percentage of the gait cycle where peak amplitude occurred.

Peak amplitude (Fig 3A) or the percentage gait cycle where peak internal oblique (sEMG) amplitude occurred (Fig 3C) did not differ between groups or with speed or inclination. The duration of activation of internal oblique was longer for older participants (F = 12.82, p<0.001; SNK: p<0.001) (Fig 3B).

#### External oblique

Peak external oblique activation did not differ between groups but was dependent on condition (F = 4.10, p<0.001) with greater activation when walking at 4km/h-10% compared to walking at each inclination at 2km/h (SNK: all p<0.05) (Fig 3D). Duration of external oblique activation was longer in older participants (F = 21.98, p<0.000001; SNK: p<0.0001) and changed with speed (F = 8.86, p<0.01) where activation was longer at 2km/h (SNK: p<0.01) (Fig 3E). The percentage gait cycle where peak external oblique (sEMG) amplitude occurred, differed between groups (F = 4.06, p<0.05) but was not changed by speed or inclination. Specifically, peak of activation occurred earlier in older participants (SNK: p<0.05) (Fig 3F).

### Kinematics

#### Spine

Results for kinematics are presented in Table 1 (mean values ±SDs) and Table 2 (comparisons). ROM at the lumbar spine (lumbar lordosis) was less (F = 27.83, p<0.000001) in all conditions for older than younger participants (SNK: p< 0.000001) but did not differ with condition.

Trunk inclination depended on group (F = 22.60, p<0.000001) and speed (F = 4.41, p<0.05) but not inclination (p>0.05); no interactions were present. Trunk inclination was greater at 2km/h than 4km/h (SNK: p<0.05) but smaller for older participants for all conditions (SNK: p<0.0001).

Pelvic rotation depended on group (F = 105.92, p<0.000001) and speed (F = 44.64, p<0.000001) but not inclination (p>0.05); group and speed interacted (F = 29.63, p<0.000001) where older participants did not change with speed (SNK: p>0.05); however, there was an increase in pelvic rotation at 4km/h compared to 2km/h in younger participants (SNK: p<0.00001). Younger participants had more pelvic rotation at both speeds (SNK: both p<0.001) and at 2km/h than older participants at 4km/h (SNK: p<0.05).

**Table 1:** Mean (±SD) of spine, hip, knee and ankle range of motion (degrees) during walking at different speeds (km/h) and inclinations (%) between young and older adults.

		*2km/h*				*4km/h*			
		0%	1%	5°	10°	0°	1°	5°	10°
*Lumbar lordosis*	Young	4.4±3.8	4.6±3.5	4.2±2.4	4.2±2.9	4.6±2.5	5.3±3.3	5.5±3.4	5.4±3.7
	Older	2.7±1.4	2.4±1.2	2.3±1.2	2.5±1.4	3.0±1.4	3.0±1.0	3.0±1.3	2.6±1.3
*Trunk inclination*	Young	2.9±0.8	2.8±1.2	2.7±0.6	2.9±1.0	2.6±0.6	2.7±0.6	2.6±0.6	2.7±0.8
	Older	2.3±0.8	2.5±0.6	2.4±0.6	2.3±0.8	2.2±0.5	2.2±0.4	1.9±0.5	1.8±0.3
*Pelvic rotation*	Young	7.6±1.8	7.8±1.5	8.5±2.5	9.6±2.6	14.2±3.4	14.2±2.8	14.2±2.6	14.9±4.0
	Older	5.3±3.0	5.3±2.8	6.0±3.3	7.3±3.4	7.2±4.1	6.9±4.4	6.7±3.2	5.6±3.0
*Hip*	Young	26.3±5.5	27.0±3.9	30.0±7.0	33.1±5.9	30.7±6.7	31.9±7.7	34.4±7.1	40.9±7.8
	Older	24.8±5.5	25.0±5.0	26.3±5.2	28.3±5.1	29.1± 5.1	29.5±4.9	31.9±5.1	33.0±5.5
*Knee*	Young	47.3±4.1	46.3±3.9	46.6±3.7	46.7±3.6	54.6±4.3	54.2±3.6	54.2±3.6	52.9±4.9
	Older	44.8±6.5	44.3±7.8	43.3±8.1	47.6±12.2	53.1±9.2	52.9±9.3	52.8±10.1	48.3±5.2
*Ankle*	Young	13.2±3.5	14.0±3.2	15.6±3.7	18.2±4.8	21.2±5.7	22.9±5.9	24.9±6.1	29.9±6.9
	Older	18.9±15.3	18.9±15.2	20.6±16.6	24.7±20.2	29.4±18.8	29.1±19.1	31.0±19.0	27.7±5.4

**Table 2:** Differences in kinematics according to age-group, speed and inclinations.

	Young vs older	Speed (2km/h, 4km/h)	Inclination (0,1,5,10°)
**Lumbar lordosis**	p<0.000001	NS	NS
**Trunk inclination**	p<0.000001	p<0.05	NS
**Pelvic rotation**	p<0.000001	p<0.000001	NS
**Hip**	p<0.001	p<0.000001	p<0.00001
**Knee**	NS	p<0.00001	NS
**Ankle**	p<0.05	p<0.00001	NS

#### Lower limb

Hip ROM was dependent on group (F = 11.96, p<0.001), speed (F = 29.06, p<0.000001) and inclination (F = 8.47, p<0.00001) with no interactions. Lower hip ROM was shown for older participants (SNK: p<0.001). Hip ROM increased when walking at 4km/h than 2km/h (SNK: p<0.00001), and at 10% incline compared to all other inclines (SNK: p<0.01).

Knee ROM was comparable between groups and changed with walking speed (F = 39.51, p<0.00001) but not inclination. Larger knee ROM was observed at 4km/h (SNK: p<0.00001).

Ankle ROM differed between groups (F = 6.49, p<0.05) and varied with speed (F = 20.16, p<0.00001), but not inclination. Specifically, ankle ROM was larger for older participants (SNK: p<0.01), and at faster speed (SNK: p<0.0001).

## Discussion

This study provides new information regarding age-related activity of the thoracolumbar paravertebral and trunk muscles during treadmill walking. Longer duration of muscle activation was generally seen in the older adult group across all muscles and walking conditions examined. However, while we expected higher amplitude activity in older adults particularly in walking conditions that might be considered more challenging, this was not consistently shown. The differential activation expected between deep and superficial multifidus fibres was arguably more notable in the older adults where superficial fibres appeared to have lower activity than in the younger group. Assessment of kinematics of the spine, trunk, and lower limbs generally confirmed reduced ROM in older participants (except at the ankle).

Our results show that the largest difference in thoracolumbar muscle activity between young and older adults is in the duration of activation. With the exception of superficial multifidus (all conditions) and deep multifidus (at 4km/h), older adults had prolonged muscle activation unrelated to where in the gait cycle peak activation occurred, the peak amplitude itself, or walking condition (speed or inclination). Whether this finding has a mechanical or metabolic fundament related to the demand of walking is difficult to speculate given our small samples; however, longer duration activity has implications for rehabilitation regarding exercise tolerance. Further study examining larger samples across wider age groups to represent potential progression of age-related change to spinal muscle activation may be beneficial. Further, known sex differences [20] in spinal kinematics [48] and muscle composition [1] should inform sub-group analyses.

Lower peak amplitude of superficial multifidus was shown for our older participants. Given our older participants used less lumbopelvic ROM in all conditions, this result seems reasonable for the multifidus fibres primarily associated with extension in maintaining an upright posture [34, 35]. Deep multifidus activity relates more to the walking condition, where older adults show earlier onset, and younger adults show longer duration at 4km/h. Further, the higher values shown for deep multifidus at 4km/h-10% may indicate an increased requirement for motion segment stabilisation in more demanding walking. There appears merit in determining what factors most influence the demand of walking for older adults, even in controlled environments such as those we employed. Interestingly, Weber et al. [25] tested spinal and lower limb muscle activity in nine young men in a ‘natural’ and a more demanding walking condition and reported superficial multifidus as the only muscle tested where activity differed between walking condition, wherein higher activity and longer duration occurred with demanding walking. Further, in examining co-activation between RA:MF (superficial) during walking, Lee et al. [10] revealed reduced co-activation with age, which may indicate an increasing contribution from (superficial) multifidus, but may instead reflect reduced rectus abdominus activity with no concomitant change in multifidus.

Lumbar and thoracolumbar erector spinae reached higher peak amplitudes in older participants, but this was not shown for thoracic erector spinae. This supports the concept that ageing influences muscles non-uniformly and relating to function [2, 3, 49]. Further, this result may mean greater age-related modifications in fibres of the lumbar than thoracic region, which is plausible as spinal curvature and posture influence muscle composition [50, 51]. In addition, our older participants walked with less lumbar lordosis, trunk inclination, and pelvic rotation than younger participants, and did not modify their pelvic rotation with speed while younger participants used more pelvic rotation when walking faster. Whether this finding represents a stiffening of the lower spine, trunk, and proximal lower limb with age concurrent with the normative degenerative cascade [52, 53] requires further exploration.

Our results for the abdominal obliques are challenging to interpret, where external oblique was influenced by age and walking condition more than internal oblique activity. The external obliques showed earlier onset and longer duration in older adults, and higher peak amplitude at 4km/h-10% than each 2km/h condition. Of the muscles tested in our study, external oblique was the only muscle to engage earlier in the gait cycle in older than younger adults. Capturing motion of the upper limbs and trunk rotation may improve our understanding of this result. However, it is plausible that the demand of the imposed walking conditions were higher for the older than younger participants, particularly in light of the narrower ROM throughout the trunk, spine and lower limb employed by the older adults. Examining self-selected ‘slow’ and ‘fast’ speeds may mitigate this influence although ensuring safety of participants at the highest inclinations remains a difficult methodological consideration.

In terms of walking as a specific activity target for thoracolumbar spinal muscles, the 4km/h-10% condition appears promising. Further, our results support the notion that faster treadmill walking (at 4km/h in our study) requires greater and more prolonged activity from thoracolumbar paravertebral muscles irrespective of inclination. Using sEMG, Anders et al. [20] showed that at higher walking speeds, amplitude curves became more phasic and differed between sexes. Whether walking has clinical significance as an intervention to modify muscle activity requires examination in longitudinal studies. Further, due to the morphological complexity of thoracolumbar muscles where intramuscular variations like fibre length exist within and between vertebral levels and regions, we recommend that intra-muscular regions be considered separately and at multiple vertebral levels to identify age-related modifications to function. New evidence is pointing the importance of specific intervertebral level to muscle composition in neurocompressive (and degenerative) conditions of the lumbar spine,[54] which age strongly influences.

Our results in small samples of asymptomatic young and older adults may serve as an early indication of differences in spinal and trunk muscle function that occurs with age. However, further investigation is necessary and the study limitations should be considered when interpreting the findings. While adequate for this descriptive observational study and given the invasive nature of the methods used, we examined relatively small, mixed-sex samples. Future studies should aim to enrol larger samples across several age groups to allow for analyses to identify when, which, and in whom, muscles express modifications over time. In attempts to homogenise methods across our age-groups, we selected two speeds that young and older adults could complete safely at the highest inclination; that these speeds best represent slow and fast for each individual is unlikely and further studies require an approach centred on self-selected speeds. As such, 4km/h-5-10% may not be the optimum walking condition to engage thoracolumbar spinal muscles for asymptomatic adults of all ages. However, in examining walking as a promising intervention specifically directed at spinal muscles, this specific condition appears a reasonable starting point. Longitudinal studies that determine the potential of a walking intervention in modifying thoracolumbar muscle composition and quality, and in relation to pain and/or spinal neuromusculoskeletal disease, are warranted and underway.
